# Identification of Global Transcriptional Dynamics

**DOI:** 10.1371/journal.pone.0005992

**Published:** 2009-07-10

**Authors:** Eric H. Yang, Richard R. Almon, Debra C. DuBois, Willian J. Jusko, Ioannis P. Androulakis

**Affiliations:** 1 Biomedical Engineering Department, Rutgers University, New Jersey, United States of America; 2 Department of Biological Sciences, State University of New York at Buffalo, Buffalo, New York, United States of America; 3 Department of Pharmaceutical Sciences, State University of New York at Buffalo, Buffalo, New York, United States of America; 4 New York State Center of Excellence in Bioinformatics and Life Sciences, Buffalo, New York, United States of America; Fondazione Telethon, Italy

## Abstract

**Background:**

One of the challenges in exploiting high throughput measurement techniques such as microarrays is the conversion of the vast amounts of data obtained into relevant knowledge. Of particular importance is the identification of the intrinsic response of a transcriptional experiment and the characterization of the underlying dynamics.

**Methodology and Findings:**

The proposed algorithm seeks to provide the researcher a summary as to various aspects relating to the dynamic progression of a biological system, rather than that of individual genes. The approach is based on the identification of smaller number of expression motifs that define the transcriptional state of the system which quantifies the deviation of the cellular response from a control state in the presence of an external perturbation. The approach is demonstrated with a number of data sets including a synthetic base case and four animal studies. The synthetic dataset will be used to establish the response of the algorithm on a “null” dataset, whereas the four different experimental datasets represent a spectrum of possible time course experiments in terms of the degree of perturbation associated with the experiment as well as representing a wide range of temporal sampling strategies. This wide range of experimental datasets will thus allow us to explore the performance of the proposed algorithm and determine its ability identify relevant information.

**Conclusions and Significance:**

In this work, we present a computational approach which operates on high throughput temporal gene expression data to assess the information content of the experiment, identify dynamic markers of important processes associated with the experimental perturbation, and summarize in a concise manner the evolution of the system over time with respect to the experimental perturbation.

## Introduction

With the advent of microarray technologies for measuring genome-scale transcriptional responses, there has been a renewed interest in using computational methodologies to study systemic responses [1]. The main motivation behind these approaches is that while the expression levels of thousands of genes have been measured, there exists a smaller subset which is representative of the underlying phenomenon being investigated. Thus the primary difference between the various algorithms that have been proposed lies in their individual hypotheses as to what comprises an informative feature of the data, i.e. a characteristic which can be used to give insight into the importance of a given gene. Commonly used methods such as ANOVA [2], t-test [3], and SAM [4] assume that genes which show changes in gene expression, across conditions or relative to a control state are relevant, with the difference between the algorithms dependent upon their associated definition of differential expression.

For deciphering the dynamics of biological responses, temporal gene expression experiments record transcriptional changes over time with the goal of establishing a broader range of co-expression characteristics [5]. It is hypothesized that temporally varying signals can provide valuable insight into the progression of the biological system in response to an external perturbation. In light of this kind of data, it is hypothesized that utilizing only differential expression may ignore a great deal of information present within a given temporal gene expression dataset. As such, a number of powerful methods have emerged that evaluate the presence of over-populated clusters composed of genes characterized by similar temporal profiles.[6], [7], [8], [9].

In this paper we hypothesize that an emergent relation between genes may be an important feature denoting biological relevance of a gene by being part of a coherent response. This hypothesis arises from a basic concept of systems biology in which the response of an organism to an external stimulus is made up of the synchronized response of a group of genes [10]. To further explore this hypothesis, we have formulated an algorithm which takes the coordination between different genes into account. In addition to the set of selected genes characterized as informative, we hypothesize that the coordination between different genes and clusters can yield information as to the overall response of the system and whether the system has undergone significant experimental perturbations, thus indicating whether the experimental result has been successful in capturing an underlying perturbation.

In this paper we extend the analysis earlier presented in [11] by demonstrating how to address three critical questions: (i) does the systemic response exhibit a characteristic underlying dynamic? (ii) what are the temporal properties of the inferred dynamic? (iii) is there a minimal set of transcriptional responses that underlie the emergence of the complex cellular dynamic? The last question is a potentially critical one as it can allow, through a reductionist approach, the identification of the minimal complexity that drives a host's response to an external perturbation. The latter is a concept that is attracting ever increasing interest [12]. We demonstrate the approach through the analysis of five datasets, a synthetic set to be used as the negative control and four case studies.

## Results

We provide first a short overview of the approach so that the reader can follow the discussion without delving into the algorithmic details which will be extensively discussed in later parts of the manuscript. The algorithm is an integrative clustering and selection algorithm. Rather than selecting genes based upon differential expression, the algorithm selects patterns (motifs) within the data based upon the over-representation of that specific pattern and its contribution to the overall response of the system. The proposed algorithm consisted of two primary steps: (i) a fine grained clustering algorithm to identify an extensive list of putative clusters, and (ii) a selection operation to determine which of the clusters are representative of the underlying response. The fine-grained clustering, based on a symbolic transformation, allows for the identification of a large number of possible expression motifs. A selection process which follows allows for the selection of the subset of most critical and characteristic responses. The combinatorial selection of the informative subset of expression motifs will be performed using a greedy and/or a global method. We have identified two metrics for quantifying deviations from homeostasis: a global metric, denoted by Δ, and a time dependent, termed the Transcription State denoted by Δ(t). Our underlying hypothesis is that only informative motifs should contribute to deviations in the metrics from homeostasis.

### Selection of Informative Motifs

#### Circadian

**Figure 1:**
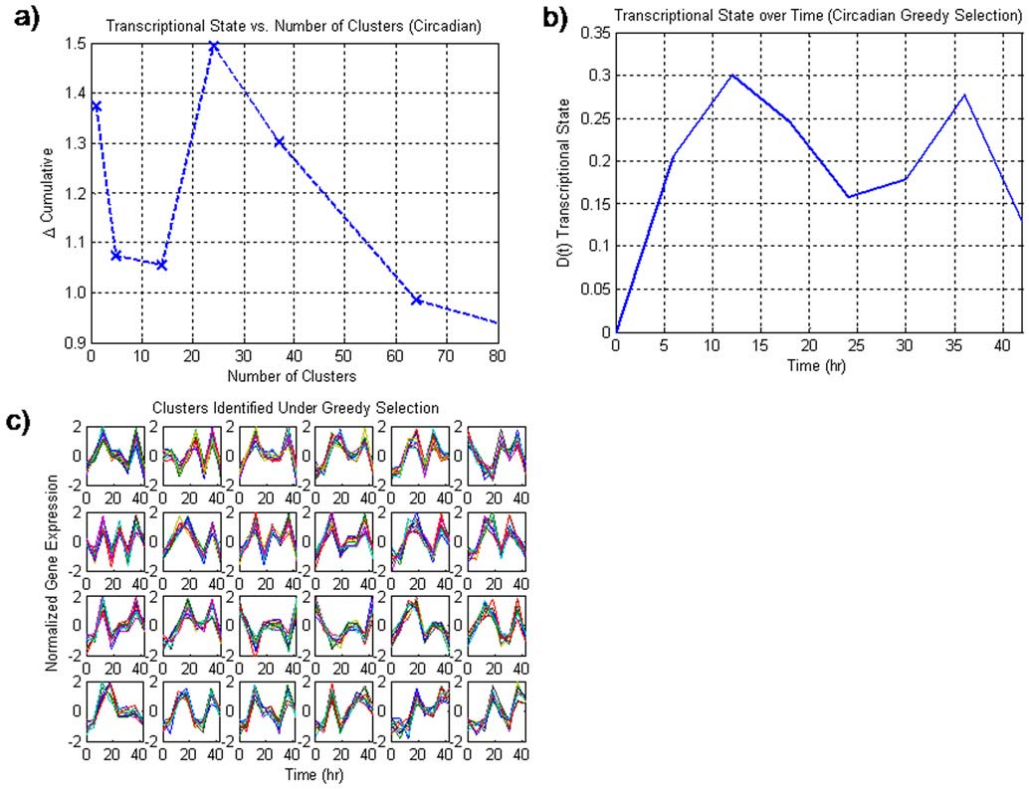
a) The transcriptional state as a function of the number of clusters selected for the circadian dataset. Unlike the null synthetic dataset, there is a maximum at an intermediate number of clusters thus signifying the incorporation of important information. b) the transcriptional state over time associated with the greedy selection. This response suggests a periodic circadian characteristic which is in agreement with the underlying data. c). The 24 clusters that were selected as informative genes. The selection of clusters which do not exhibit 24 hour periodicity may be due to the suboptimal greedy selection.

In the case of the circadian dataset, the application of the greedy selection **(** **Figure 1a** **)** demonstrates that the incorporation of additional motifs past a certain maximum (24) does not introduce any new information as indicated by the decrease in the transcriptional state. Associated with this maximum in the transcriptional state, max(Δ(t)), is the plot for the transcriptional state Δ(t) **(** **Figure 1b** **)** vs. time as well as the twenty-four clusters associated with the selection **(** **Figure 1c** **)**. In **(** **Figure 1b** **)**, we are plotting the deviation of the set of informative genes from the control state at time 0. The interesting feature of this plot is that the transcriptional state appears to change in a periodic manner with period spikes present at 12 and 36 hours, accounting for a 24 hour periodicity within the data. Thus, while the proposed algorithm has utilized no specific information about the periodicity of the data, it was still possible to discern the underlying pattern within the data. This is in contrast to the original analysis utilizing the Lomb-Scargle algorithm which assumes periodicity. The motifs which were selected by the algorithm are presented in **(** **Figure 1c** **)**. While not all of the selected clusters have a clear 24 hour periodicity, the majority of the clusters do have a 24 hour oscillatory behavior.

#### Acute Administration of Corticosteroids

**Figure 2:**
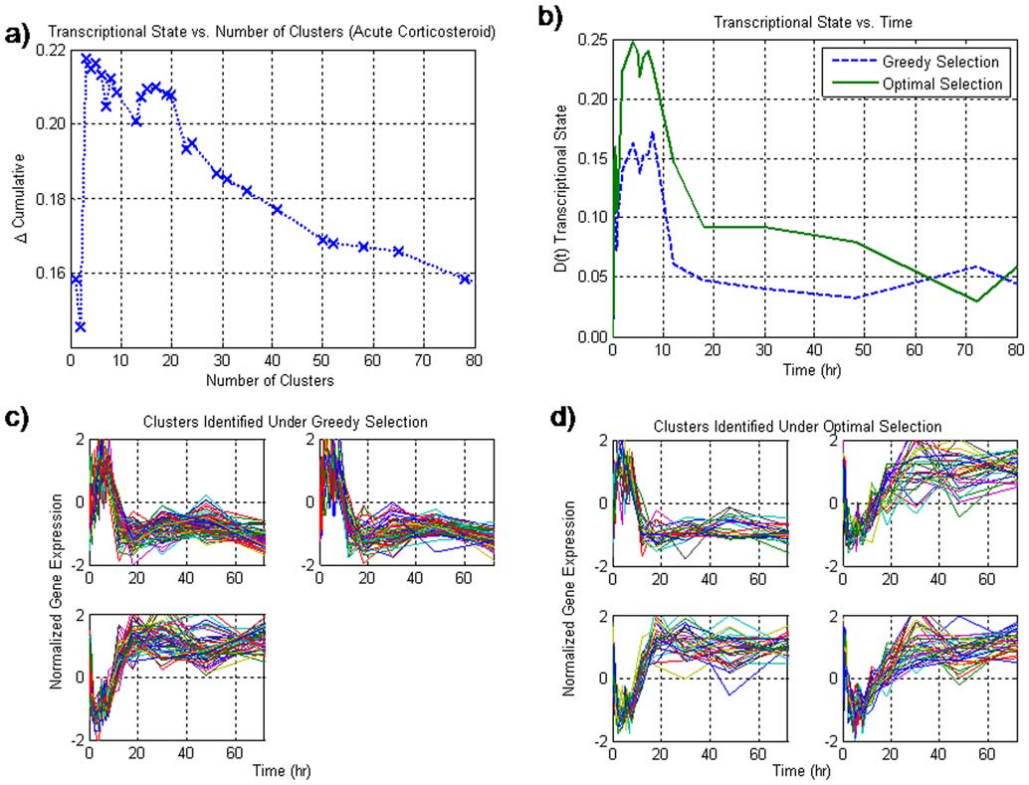
a) The transcriptional state as a function of the number of clusters selected for the acute corticosteroid dataset. Unlike the null synthetic dataset, there is a maximum at an intermediate number of clusters thus signifying the incorporation of important information. b) the dynamics of the transcriptional state over time for the two methods for selection. In this graph, it is clear that the overall characteristics of the dynamics do not change. However, the dynamics associated with the optimal selection is much greater than that of the greedy selection. c) the three clusters assoicated with the greedy selection. All of these clusters appear to have a similar deviation away from baseline and a return back to baseline. d) the optimal selection yields qualitatively similar profiles despite the fact that there is no overlap between the two sets. In this case as in the greedy selection, there is a deviation away from baseline and a return back to baseline.

In the case of the acute administration of corticosteroids the maximum deviation of the transcriptional state occurs at some intermediate level **(** **Figure 2a** **)**. Beyond this point, as more motifs are added, there is a decrease in deviation exhibited by the transcriptional state. In a similar fashion to the circadian dataset, this decrease in the value of the transcriptional state is associated with the incorporation of motifs which are either very similar to ones that have already been added and thus add no information, or are sparsely populated and thus not significant. The progression of the transcriptional state of the acute corticosteroid dataset shown in **Figure 2b**, comparing the greedy and optimal section as well, and is comprised of a deviation away from the baseline as the drug is injected into the system and activates the transcriptional machinery, and a return back to baseline as the drug is cleared from the system. This overall systemic response is similar to the response predicted via the indirect effect model [13]. This suggests that this transcriptional state is an accurate surrogate to describe the systemic activity of the drug. The 212 genes divided into 3 clusters that are associated with this transcriptional state are given in **Figure 2c and 2d** for the greedy and optimal respectively, and interestingly enough the extracted genes essentially follow the same profile with a deviation away from the baseline state and then a return back to the original baseline state.

#### Chronic Administration of Corticosteroids

**Figure 3:**
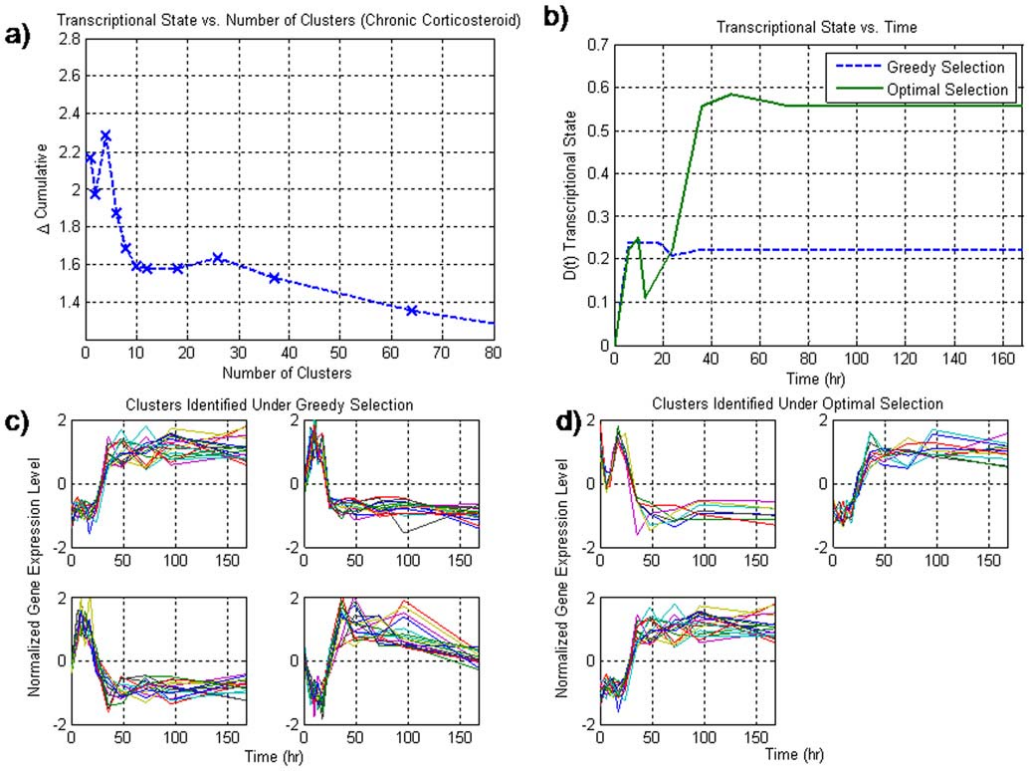
a) The transcriptional state as a function of the number of clusters selected for the chronic corticosteroid dataset. Unlike the null synthetic dataset, there is a maximum at an intermediate number of clusters thus signifying the incorporation of important information. b) the dynamics of the transcriptional state over time for the two methods for selection. What is evident is that not does the transcriptional state show a larger deviation, but the two wave effect is also more pronounced when utilizing the optimal selection. c) the four clusters associated with the greedy selection. There seems to be two distinct profiles associated with these clusters consistent of a transient regulation and a sustained response that is active after an initial delay d) the optimal selection yields 3 similar profiles.

Under a chronic administration of corticosteroids, we identify a similar level of over-representation in the population dynamics as in the acute administration of corticosteroids. However, while the level of correlation associated with this dataset is not as low as that of the acute corticosteroid dataset, it is evident that there exists a subset of motifs that do show a significantly non-exponential characteristic. During the greedy selection process, we see a response which is qualitatively similar to that of the acute corticosteroid case as well as the circadian dataset in which a maximum is reached at an intermediate number of clusters (4), after which there is a decline, **Figure 3a**. Again, there is a visible increase in the deviation away from the baseline state as more clusters are added rather than a strict decrease. Therefore, this dataset illustrates both a significant perturbation to the system as well being informative. Running the greedy selection upon this dataset yielded four different motifs with 67 probe sets associated with them **(** **Figure 3c** **)**. These motifs follow two distinct patterns. The first pattern is an initial deviation away from the baseline state, and a return back to baseline, whereas the second motif represents a sustained up-regulated response. This response is unusual because it is different from those observed under the acute case where there all of the profiles had similar characteristics albeit the profiles were anti-correlated with a few being up-regulated and the others being down-regulated. With chronic administration, it appears that the responses are very different because one of the profiles shows diminished response despite a sustained input of corticosteroids, whereas other profiles show a sustained response due to the infusion.

The transcriptional state for this drug administration shows a similar dynamic, in which there is an initial deviation, and a slight return to baseline, before a second sustained response takes over **Figure 3b**. This response mimics the response that is associated with sustained corticosteroid administration in which there are some phenomenon which are transient such as the immuno-suppressive effect of corticosteroids [14], whereas, although not exclusively, the metabolic side effects such as muscle wasting appear to be sustained. Filtering the dataset for over-represented motifs led to a reduction from 177147 possible motifs to 26 over-populated motifs. From these 26 over-populated motifs 3 were selected as encompassing the optimal set of responses that best describe the dynamics of the system. The optimal selection identified motifs which showed sustained activation as well as a short-term response followed by return to baseline exemplifying a tolerance mechanism associated with corticosteroids [15]. Likewise the transcriptional state associated with this set of motifs **Figure 3b** **(solid)** appears to show a more pronounced two-stage effect in which there is an initial deviation away from homeostasis, a slight return, after which a secondary effect takes over. Thus, the small decrease that was evident in the greedy selection was not an artifact of the data, but rather some intrinsic event.

#### Burn Injury

**Figure 4:**
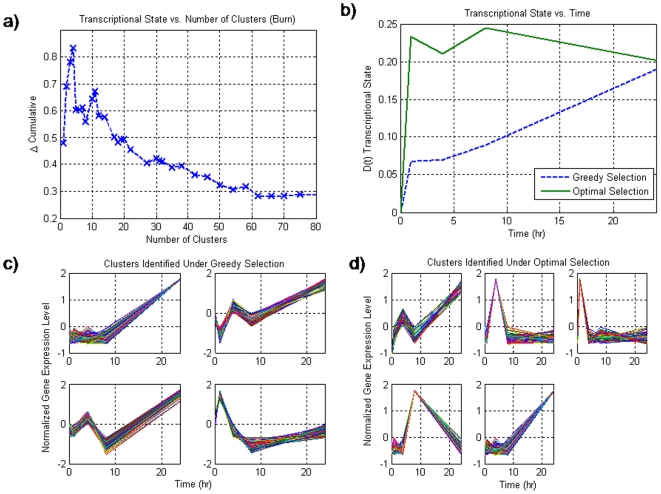
a) The transcriptional state as a function of the number of clusters selected for the burn dataset. Unlike the null synthetic dataset, there is a maximum at an intermediate number of clusters thus signifying the incorporation of important information. b) the dynamics of the transcriptional state over time for the two methods for selection. What is evident is that not does the transcriptional state show a larger deviation, but the two wave effect is also more pronounced when utilizing the optimal selection. c) the three clusters associated with the greedy selection. In the greedy selection there appears to be a two wave effect. d) unlike in the previous datasets, there is a significant difference in the response from the optimal selection vs. that of the greedy selection. Under the optimal response, there appears to be a two wave response, as well as four distinct activation events at four different time points which may represent the activity of a cascade of signaling events in response to a significant thermal injury.

The burn injury dataset (GDS599) yielded 4 clusters with 281 probes under the greedy selection **(** **Figure 4c** **)** and 5 clusters with 307 genes under the optimal selection **(** **Figure 4d** **)**. The global dynamics exhibits a two-wave response **(** **Figure 4b** **)** consistent with the notion of genetic reprogramming as previously documented in vivo and in vitro [16], [17]. As with the other datasets, under the greedy selection, we find that there is an intermediate number of clusters which yield a maximum deviation in the transcriptional state, after which the inclusion of additional clusters appears to diminish the appearance of the change between the different states **(** **Figure 4a** **)**. The optimal selection was conducted upon a subset of 10 clusters all of which had at least 49 genes within them.

The profiles associated with the four clusters can be described as an early up-regulation event which returns back to a different state, two bi-phasic responses which contain genes which spike at two different time points, and finally a late up-regulatory event. In contrast to the result of the greedy selection, the optimal selection shows a clear progression in the activation of different genes. In the optimal selection, our first cluster shows a similar bi-phasic response as was selected under the greedy selection, whereas our other clusters essentially show spikes at different time points, which indicate a cascade of events occurring in sequence, with spikes occurring at different time points indicating a short period of time when specific stages in a particular cascade are active. Unlike the corticosteroid datasets, the optimal selection vs. the greedy selection yielded some clusters which were qualitatively different, specifically, the appearance of the gene expression profiles which spiked at different time points.

However, through the examination of the transcriptional state, we are able to draw a link between the two results. It is observed that the burn injury appears to have an initial deviation as the organism responds to the original stimulus. Then after the cessation of the initial response of the system, there is a slight return to the baseline at hour four. However, in both cases, there is a massive secondary event which occurs that drives the system either to a new steady state as suggested in the case of the optimal selection, or uncontrollably in the case of the greedy selection. Therefore, while the transcriptional state of the system from hours 0-8 appears to be consistent, the final response at 24 hours appears to be different. Because of the inconsistencies of the burn dataset, numerous questions arise, specifically whether the inconsistencies between the two different selection methods represent an artifact within the algorithm itself, or whether there is some relationship between the two different results, which if resolvable may be more indicative of the underlying biological response, as well as aid in understanding the nature of the differences between the selection techniques.

## Discussion

**Figure 5:**
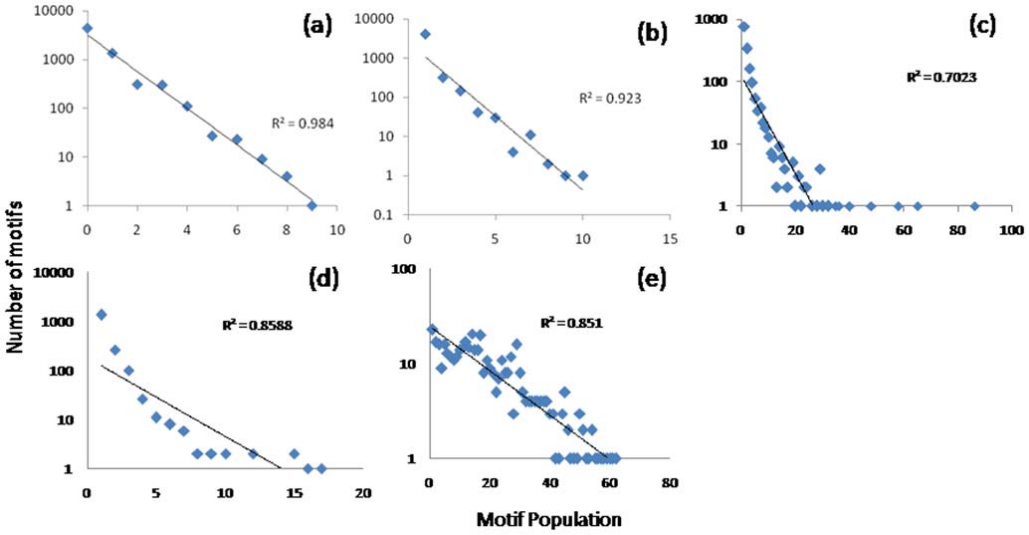
A plot indicating the number of clusters with a given size. In this plot, a) The Random dataset exhibits a high correlation with the theoretical exponential distribution (Graphs are Log-Normal) b) The circadian dataset exhibits a similar response suggesting that there is minimal perturbation. The last three datasets c) acute d) chronic e) burn all exhibit a significant deviation, especially in the tail region suggesting that the over-represented motifs occur at a rate greater than would be suggested by chance.

**Figure 6:**
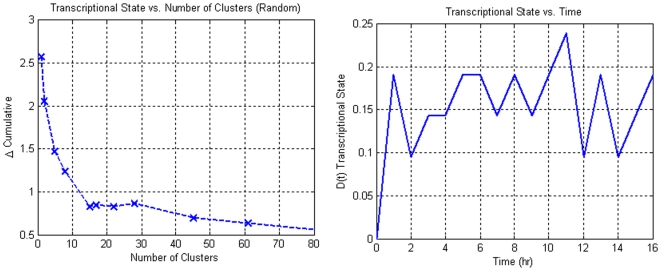
The transcriptional state as a function of the number of clusters selected for the synthetic null dataset. From this response, it is evident that with the incorporation of additional clusters adds noise into the system. Thus, no real information is present within the system.

The micro-clustering performed has allowed us to identify a large family of clusters. Depending on the nature of the data sets, we expect **(** **Figure 5** **)** an over-representation of certain cluster sizes which are indicative of coordinated responses that cannot be solely explained by random events. When evaluating the population dynamics of the different datasets, it was observed that the circadian dataset showed a population distribution very similar to that of the null dataset. However, when performing the greedy selection on the null dataset **(** **Figure 6** **)** and the circadian dataset **(** **Figure 1** **)**, we can see a clear difference between the progression of the transcriptional state of the null dataset and that of the circadian dataset. For the null dataset **(** **Figure 6** **)** we see that the maximum deviation from the baseline occurs when a single cluster has been added. The incorporation of additional clusters on the other hand serves to decrease the deviation in the transcriptional state. Thus, it appears that the incorporation of additional patterns into an informative set only serves to add “noise” into the system rather than the incorporation of additional information. Therefore, despite the fact that the population dynamics between the circadian dataset and the null dataset appear similar, this does not discount the fact that significant coordination between the different clusters exist.

### 

#### Optimal Selection

**Figure 7:**
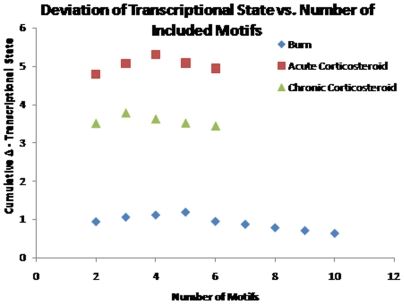
The optimal number of clusters in the three datasets is evaluated parametrically. It appears that after a certain point, the introduction of additional clusters appears to decrease the transcriptional state.

**Figure 8:**
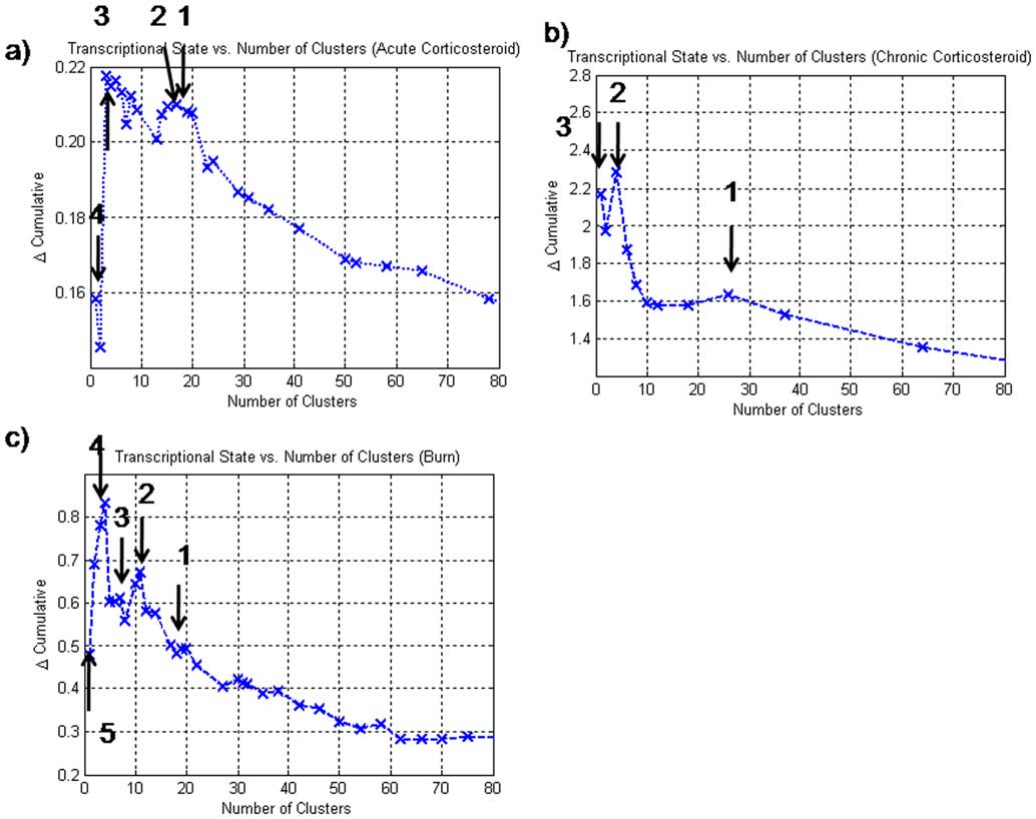
Clusters identified under the optimal selection appeared to be located near peaks that were associated with local maxima in the greedy selection. This suggests that one may be able to reduce the set to be considered for the optimal solution by looking at these peaks only.

One of the difficulties associated with the optimal selection of motifs lies in the combinatorial nature of the problem. Thus, even after eliminating the large number of motifs via their population, the combinatorial problem is not eliminated, only mitigated. Adding to this issue is the fact that the problem must be solved parametrically. Currently, we perform an exhaustive search on all possible combinations of m optimal motifs from a base population of M. For the burn dataset, there were only 10 motifs which were over-represented. Thus, parametrically exploring all possible cluster sizes was possible. However, for the two corticosteroid datasets this was not the case. In both of the corticosteroid datasets, we solved the problem parametrically for m<7. By plotting the progression of the cumulative transcriptional state for the burn dataset **(** **Figure 7** **)** we see a simple trend, where a maximum is reached at an intermediate number of clusters, and then there is a smooth decline after this point has been reached. For the two corticosteroid datasets, a similar response is observed, with an intermediate number of clusters reached before the incorporation of additional clusters decreases the expense metric. From the response of the burn dataset as well as the response of the two corticosteroid datasets, we hypothesize that the maximum number of informative motifs has indeed been obtained. After the maximum deviation has been reached, we hypothesize that there is a smooth reduction in the aggregate transcriptional state due to the addition of similar gene expression profiles. This is seen in the greedy selection as well, in which after a certain point, we see a consistent decline in transcriptional state. In an alternative strategy, we look at the performance of the greedy selection and observe where the globally optimal motifs lie. In **Figure 8**, the clusters that have been optimally selected have been associated with the cluster vs. transcriptional state plot from the greedy selection algorithm. The arrows in **Figure 8** indicate the point at which a cluster which was selected under the optimal selection was selected via greedy selection. The interesting trend which we see is that the clusters that are identified via the optimal selection are usually located around local maxima associated with the greedy selection. We hypothesize that during the greedy selection strategy, motifs that are similar in shape decrease the overall value of the transcriptional state because no new information is being incorporated, whereas the introduction of a new pattern will cause a significant increase in the transcriptional state. Therefore, there appears to be a direct link between the dynamics of the cluster vs. transcriptional state plot and the location of representative patterns. Furthermore, it appears that the optimal selection directly selects for a set of “representative” patterns within the data, due to their correspondence with the local maxima.

One issue of concern for us is the reliance upon over-represented motifs when conducting the optimal selection. Because we are using an exhaustive enumeration of motifs, it is critical for us to identify a subset of possibility meaningful motifs. However, in the case of the circadian dataset, we are unable to identify over-represented motifs, and would thus have to run it upon all of motifs in the dataset. This combinatorial problem has not been addressed in the current algorithm, but can be addressed by more complex heuristics that can be implemented in the future.

The proposed algorithm represents a different method for processing high throughput temporal gene expression data. Rather than assessing the importance of a single gene on a case by case basis, we instead propose examining the importance of a specific pattern. Furthermore, the importance of this pattern is evaluated within the context of its contribution to an inherent underlying dynamic which is not known *a priori*. Associated with this underlying dynamic, are dynamic signals whose activity show important correlations with the underlying phenomena being investigated. In the corticosteroid datasets, the dynamic response under the acute case mimicked the response predicted by the indirect effect model in which the dynamic showed a time lag before the maximal activity was reached, and a decline as the drug was cleared from the system. In the chronic corticosteroid case, we saw profiles that corresponded well with the observations that some corticosteroid responsive phenomena were transient exhibiting a significant tolerance mechanism, whereas other corticosteroid responsive phenomena such as muscle wasting was sustained. Finally in the case of the burn dataset, our profiles appeared to illustrate the impact of a signaling cascade with significant genes turning on and off in sequence, signaling the short-term progression of pathways the organisms uses to respond to the severe injury.

## Materials and Methods

### Gene Expression Data

We have structured a compendium of transcriptional responses in order to elucidate the insights of the overall approach. A synthetic dataset is created where values are drawn from a N(0,1) distribution in order to illustrate basic properties of the calculations. In addition 4 experimental dataset are evaluated and the raw data can be found in Gene Expression Omnibus database [18].

The first experimental dataset, accession number GDS1629 [19], is a circadian dataset obtained from a rat superchiasmic nucleus (SCN), and represents a system in which there should be significant patterns within the data, even though there is no significant outside perturbation. This dataset was selected to determine if it was possible to distinguish the difference between a real dataset that did not undergo a significant perturbation and random responses. This dataset consisted of cells isolated from the SCN tissues obtained from adult Long Evans Rats. These cells were then synchronized for 48 hours prior to the experiment through media replacement before being harvested every 6 hours from 0 to 42 hours for RNA extraction. Therefore, we can investigate the progression of gene expression of these cells independent of outside stimuli which may be propagated through endocrine or neurological signaling.

The second dataset, accession number GDS253 [20], records liver transcriptional responses in adrenaelectomized rats undergoing a bolus injection of corticosteroids. In this experiment, 44 rats were injected with a methylprednisolone at a dose of 50 mg/kg. At 17 individual time points [0, 0.25, 0.5, 0.75, 1, 2, 4, 5, 5.5, 6, 7, 8, 12, 18, 30, 48, 72 hrs], the livers were harvested from the rats, and the gene expression was obtained via the Affymetrix RG-U34A array which consisted of 8799 probe sets.

In this experiment, a significant, yet reversible, perturbation has occurred to the system such that there should be a clear deviation away from the baseline case followed by return to the control state.This case was selected to validate the fact that the presence of a significant perturbation is visible along with the non-randomness of the dataset. This dataset has the added advantage of having a well-characterized mechanism which allows for the assessment as to whether the temporal variations in the transcriptional state have meaning with respect to the underlying biological phenomenon. Given the number of time points associated with this dataset, this will be the only dataset which was run with piece-wise averaging. Thus, this dataset was run with a piecewise averaging of 2, such that adjacent points are averaged to obtain a single data point. Because of the fact that 17 time points do not divide evenly into 2, this dataset was extrapolated to 18 time points with the final time point occurring at 80 hours.

The next dataset, accession number GDS972 [21], consists of a similar animal model in which a low level infusion of corticosteroids is taking place. In this experiment, methylprednisolone was administered at a rate of 0.3 mg/kg/hr over 168 hours via an Azlet osmotic pump. Over the course of time experiment 44 animals were sacrificed at 11 time points [0, 6, 10, 13, 18, 24, 36, 48, 72, 96, 168 hrs] to obtain the dataset. Unlike the previous experiment, the microarray platform for this dataset is the RAE230A, which consisted of 15923 probesets.

The final dataset which is evaluated is listed under accession number GDS599. This dataset represents a serious cutaneous burn administered to a rat over 30 percent of the skin. After the administration of the burn, the livers were harvested at 5 individual time points [0, 1, 4, 8, 24 hrs], and the gene expression data was obtained using the RG-U34A microarray. Unlike the corticosteroid datasets in which there is a single reversible perturbation to the system, this final dataset represents the induction of a complex series of events in response to the severe injury. Thus, this dataset will be used to investigate the ability of the algorithm to identify significant and salient changes within the system in response to a complex phenomenon.

### Fine-grained clustering

The preliminary step, i.e., the fine grained clustering operation, divides the temporal expression data into a large number of clusters in which the similarity between the different expression profiles in a cluster is expected to be very high. As such, any clustering algorithm could in principle accomplish this first task. However, we have elected to explore the basic principles of the HOT SAX representation [22] which transforms the time series in to an appropriate sequence of symbols. Thus every signal is represented as a finite sequence of symbols which is subsequently hashed to a unique scalar identifier. Time-courses that “hash” to the same value are assumed to belong to the same cluster.

**Figure 9:**
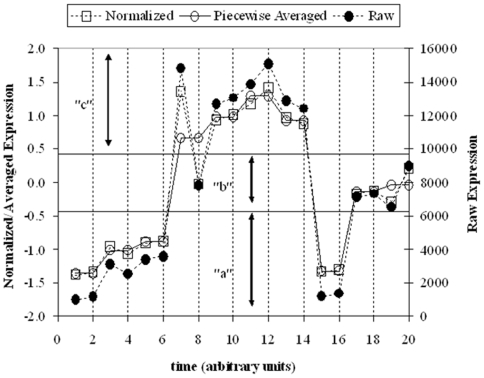
The process of converting a temporal gene expression profiles into its symbolic representation. In this figure, a randomly generated signal is discretized with a piecewise averaging parameter of 2 with 3 equiprobable partitions.

In order to emphasize the role of the shape of the responses the data is first z-score normalized such that all of the expression profiles are of the same magnitude:
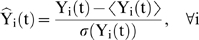
(1)This converts each of the signals to have a mean of 0 and a standard deviation of 1. Subsequently the expression value at each time point is assigned a symbol based on an appropriate equiprobable partitioning of the normal distribution **Figure 9**. A critical property relates to the number of equiprobable domains the Gaussian is divided into [22]. If necessary, the time series is also piece-wise averaged in order to reduce the size of the signal, in which case, the time horizon t = 1…T, is divided into w segments. Therefore, the original expression signal undergoes two transformations: first it is z-scored and subsequently transformed in a sequence of symbols:
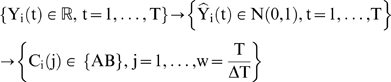



The set AB defines the so-called “alphabet” which is a set of symbols with cardinality equal to the number of equiprobable partitions of the Gaussian curve.

After a gene expression profile has been converted into a sequence of symbols, the sequence is converted into an integer through the use of an appropriate hashing function. Following the formalism of [22] we evaluate the hash value based on:
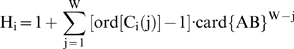
(2)The ord[•] (ordinal) operation maps the partition in which a given time point is found into a integer. The ordinal operator maps the symbol “a” to 1, “b” to 2, etc. Thus each gene expression profile Y_i_(t) hashed to unique identified, H_i_, and each such identifier corresponds to a cluster. By definition there are a finite number of hash values, which would correspond to the maximum possible number of clusters in the data. From (2) it is evident that the number of possible values for H_i_, and therefore clusters, is related to the length of the signal and the number of possible symbols which the gene expression profile has been broken up into. The use of the equiprobable distribution for the discretization step is important because signals will be assigned to the different clusters with equal probability, provided that the signals were randomly generated via an N(0,1) distribution.

Because of the underlying equiprobable distribution associated with HOT SAX, randomly generated expression profiles will be assigned different hash values with equal probability. Because of the equi-probable assignment of hash values with respect to randomly generated data, the population of a given cluster can be modeled via a Poisson process. However, in the case where there exists approximately the same number of possible hash values as genes to be hashed, this Poisson distribution can be modeled as an exponential distribution[23]. Thus, in the case of a null synthetic dataset comprised of randomly generated expression profiles, the probability that a given cluster has a population of N can be approximated via an exponential distribution.

#### Evaluation of Significant Perturbations

To evaluate whether a significant perturbation exists within the data, the hash-based clustering is run on the experimental data and a distribution of cluster membership is obtained. This is compared to a synthetic null dataset of the same size in terms of the number of genes and the number of time points generated from the random data with the same number of time points and genes as the experimental dataset. A standard permutation analysis is performed for estimating the statistical significance of a result [24]. We then determine the probability that a random trial will have a lower R^2^ correlation to the exponential distribution than the real data. From this random trial, the p-value is calculated, as p-value = Prob(R^2^ in random<R^2^ in real) to establish the confidence that the data is non-random. We expect that if there is a non-significant stimulation within the experiment the p-value should be quite high, and if there is a significant simulation within the system that the p-value should be quite low i.e. statistically significant, suggesting that indeed the system deviates reliably from the hypothetical exponential distribution.

The behavior of HOT SAX to randomly generated data thereby allows us to select the parameter AB in a systematic manner. In a real dataset, it is hypothesized that significant coordination will occur, and therefore, the performance of the hashing operation should show deviations from the theoretical exponential distribution. Thus, the HOT SAX algorithm should be run on a given dataset where AB is varied parametrically, and the AB which corresponds to the lowest correlation to the null response will be chosen as the optimal AB.

#### Selection of Characteristic Transcriptional Responses

The majority of approaches for analyzing time course gene expression data are based on the fundamental premise that over-populated motifs are indicative of significant events and thus searches for them as the main priority [9]. Our leading hypothesis extends this powerful concept and aims at the selection of a sub-set of characteristic responses, i.e., a sub-set of hash values and the cluster (motifs) they correspond to, based on the premise that hidden within the maze of responses that are recorded is a small(er) family of probe sets that exhibit clear deviations as the process of monitoring the system progresses in time. The assumption is that only the portion of the genome that is affected by the specific perturbation ought to comprise that critical ensemble of intrinsic responses. Thus the key issues now become: (i) how to assess that a non-trivial change has taken place, and (ii) how to identify the critical sub-set of responses that make up that non-trivial change.

In order to address these two issues we introduce a term which we denote as the “transcriptional state”. The transcriptional state is a metric, which quantifies the deviation from a control. The control state can be arbitrarily defined, since we are interested in deviations and not absolute values. We assume that the “control” state corresponds to time t = 0, i.e, right before the systemic perturbation, if any. The baseline state is defined as the distribution of expression values of a set of genes at the control state. To quantify the deviation from this baseline state, the difference in the distribution at any future time t and the control state is evaluated. To do so, we make use of the Kolmogorov-Smirnov (KS) Statistic [25]. The KS statistic represents a simple method for quantifying the difference between two distributions and was selected over other methods, such as the Shaprio-Wilks test [26], due to its ability to handle arbitrary binned distributions. It must be emphasized that the selection of a specific test is not binding and any effective measure for comparing two distributions can be employed. However, what is important is the ability to compare arbitrary distributions because there is no guarantee that the distribution of expression values of a set of genes will conform to a given named distribution.

**Figure 10:**
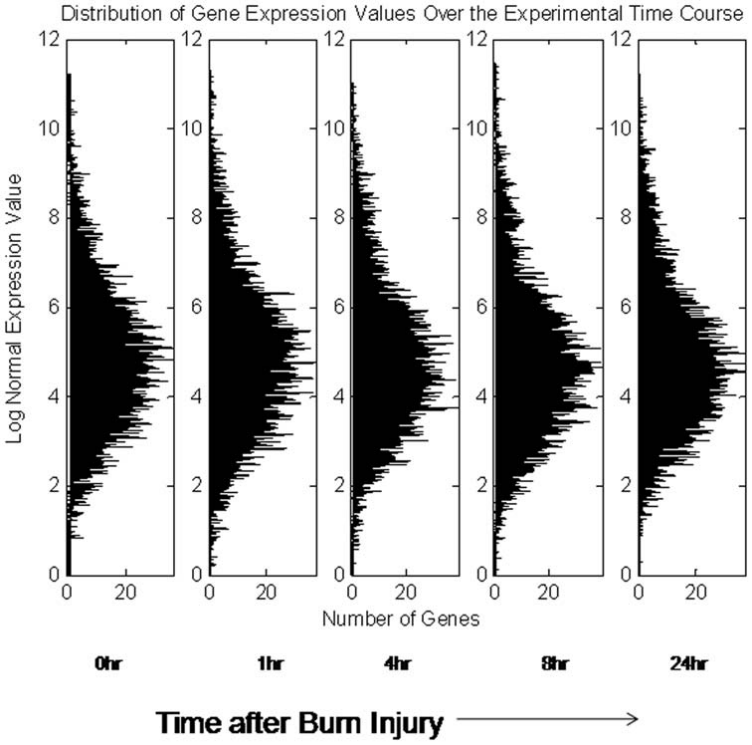
In any given gene expression experiment, the distribution of expression values at a given time point is relatively consistent due to the large amount of irrelevant gene expression profiles measured. The task is to therefore identify a subset of genes such that the shift in an organism's state as it responds to the external stimulus is maximized. This plot depicts the distribution of expression values for the burn injury data. The plot is a modification of the one presented in [27]

While it has been shown that if one takes a large enough set of genes the distribution of values is expected to follow the log-normal distribution [27], **Figure 10**. However, the distribution of expression values corresponding to the genes that exhibit the largest sensitivity to the experimental perturbation will not. In order to evaluate the KS metric the Cumulative Distribution Function (CDF) of expression levels at a given time point is determined and is compared with the corresponding CDF of the control state. For a temporal experiment this needs to be repeated over time. Since the metric is defined over binned distribution, a number of bins, B, also needs to be specified. For calculating the CDF, we have assumed that the number of bins B equals the number of genes selected under a given iteration.
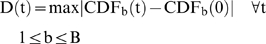
(3)


The sequence Δ(t) is defined as the transcriptional state of the system as it quantifies the level of transcriptional deviation from a control state. In order to evaluate a time-independent metric of the difference between two distributions any norm can be used on Δ(t). We opt for the simplest evaluation using the L_1_-norm. The use of the L_1_ norm, quantifies the deviation over all time points during the duration of the experimental protocol. Therefore, the scalar quantifying the difference between the two distributions is defined as:
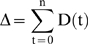
(4)Δ allows one to evaluate how by how much the distribution of expression values deviated, over time, from a control state, which as previously mentioned is considered to be the state at t = 0.

Having defined a metric quantifying the deviation of the current state from a control, the selection step of the algorithm identifies a subset of motifs composed of genes whose transcriptional state is responsible for the maximum deviation from the control state Two interesting properties of the transcriptional state are worth exploring further. The first relates to the changing character of the transcriptional dynamics, i.e., the deviation from the control, as more and more clusters are added. Based on the hypothesis that the totality of the transcriptome hides the informative components of the response, one should expect (see **Figure 10**) that the deviation from the control should be minimal if the entire data set is used. Therefore, by assessing Δ after a certain subset of clusters has been selected, we can measure whether the incorporation of additional clusters is not adding information through the inclusion of clusters highly correlated with existing ones, or whether the additional clusters are making the deviation from the baseline more evident. In the case of the null dataset, we expect that there would not be significant coordination between the clusters. Thus, the expected result is that the maximum Δ would be at a single cluster, and that Δ would be essentially a decreasing function as more clusters were added. This would mean that there are no significant anti-correlative patterns within the data, and that as more patterns are considered, one is not adding new information. In a dataset exhibiting a coherent reaction in response to a stimulus, the expectation is that Δ would reach a maximum at some intermediate value, as a population of patterns which represent the underlying dynamics has been isolated. The second characteristic is related to the dynamic progression, Δ(t), over time as it represents the temporal deviations and we hypothesize that it acts as a surrogate of the intrinsic dynamics of the system. Thus, Δ(t) summarizes the response of the system, and can be potentially useful for deriving a cellular dynamics response model.

However, the identification of an informative subset of motifs represents a difficult combinatorial problem. Given that the number of possible motifs is defined as AB^T^, where T is the number of piecewise averaged time points, the number of combinations that need to be evaluated is computationally intractable. To compensate for the combinatorial nature of the problem, we propose two different methods for carrying out the selection of informative motifs. The first method which we propose is the use of a greedy algorithm [28] in which motifs are added in the order of their population, and the transcriptional state will be evaluated each time an additional motif is added. The set of motifs which yield the greatest value for the transcriptional state will be selected as the optimal set. The justification of utilizing a greedy selection algorithm is that genes that are part of more highly population motifs have been hypothesized to be more important as compared to genes which are part of more sparely populated motifs [29].

The advantage of utilizing a greedy selection lies in the fact that the combinatorial nature of the problem is ignored at the cost of finding a sub-optimal though possibly “good enough” solution.

An alternative method for selecting an optimal subset of motifs is to limit the set of motifs that will be considered. Thus, rather than considering a superset of AB^T^ different motifs, we will limit our evaluation to over-populated motifs. Thus, by limiting the superset to only the over-populated motifs, the number of combinations that must be evaluated is decreased to a more tractable number. To perform this reduction, we define an over-populated motif as a motif whose population is greater than would be expected if the HOT SAX hashing algorithm were performed upon a randomly generated dataset which comprises the same number of probe sets and time points as the dataset being evaluated. After the initial set of motifs has been filtered, we generate all possible combination of motifs and evaluate them for the value of their transcriptional state, and like in the greedy selection, the set of motifs which yield the maximum transcriptional state will be identified as the informative subset. The advantage of utilizing this method is that unlike the greedy algorithm we can be sure that the set of motifs is indeed optimal rather.” However, while this filtering step has eliminated a large number of combinations that need to be considered, it still requires the evaluation of a large number of possible combinations and thus is computationally expensive.

### Assessing the Informative Nature of a Transcriptional Experiment

**Figure 11:**
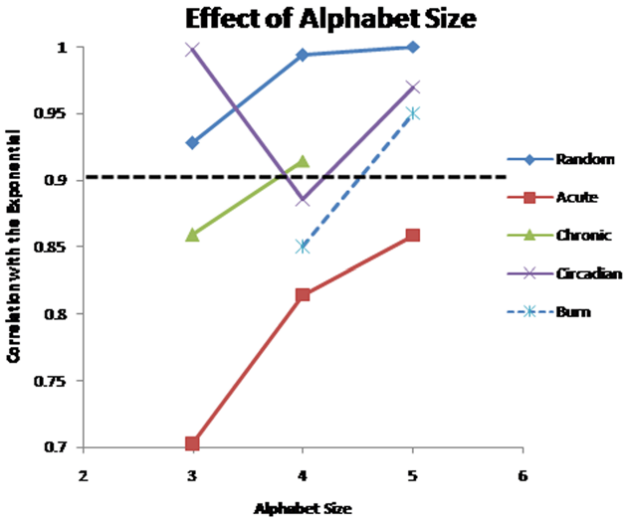
Population distribution of the different datasets as the alphabet size is altered. The distribution of hash values for the circadian and randomly generated datasets, appear to be drawn from an exponential distribution, whereas the chronic and circadian datasets are not. We select the alphabet size that shows the lowest similarity with the exponential distribution as the optimal. The burn dataset is evaluated for only alphabet sizes 4-5, because with an alphabet size of 3, the number of clusters (243) is too small for the exponential approximation to be used, whereas in the chronic dataset alphabet size of 5 was not considered because 49 millions clusters needed to be evaluated which is several orders of magnitude greater than the 15 thousand genes being evaluate.

Because the result of the HOT SAX algorithm itself depends upon the selection of the alphabet, AB, we further investigated how well the datasets correspond to the underlying exponential distribution as the value of AB is altered parametrically. Therefore, the previous evaluation as to whether a dataset consists of a significant perturbation was re-run by varying the AB parameter from 3 to 5 which are commonly used alphabet sizes. In **Figure 11**, we depict the implications of varying the alphabet size. Under all cases, the corticosteroid and the burn datasets exhibit a much greater deviation from the underlying exponential distribution than the circadian and random datasets, as expressed by the R^2^ value of the corresponding fit to an exponential. Furthermore, it was observed that by setting the AB parameter to 3, one is able to maximize the deviation away from the exponential distribution for the corticosteroid datasets, whereas an AB parameter of 4 maximized the deviation away from the exponential distribution for the circadian dataset and the burn datasets. Furthermore, the response of the randomized datasets stayed at an R^2^ = 0.9 or higher under all alphabet sizes, thus establishing a cutoff for us to determine explicitly whether the dataset represents a significant perturbation or not.

From this behavior, we hypothesized that the selection of the AB parameter should aim to maximize the observed deviation. Thus to conduct the selection of informative motifs, we have elected to utilize the results from the parametric evaluation and select an AB of 3 for the corticosteroid datasets, and an AB of 4 for the circadian and burn datasets. Despite the fact that the circadian dataset does not illustrate any defining perturbation, the selection of AB of 4 allows us to maintain a consistency

For the optimal alphabet size, we evaluate the population distribution of the individual datasets. In **Table 1**, we list the number of theoretically possible motifs as well as the number of non-zero motifs which we have identified in each dataset. The cluster size distribution for all five data sets were evaluated and as previously speculated the synthetic and circadian datasets conform closely to the expected exponential distribution **Figure 5**. For these two datasets, this was the expected result because in neither case did we assume that a significant underlying perturbation has occurred to the system. In contrast, the two datasets corresponding to an administration of corticosteroids exhibit a significant deviation from this exponential distribution as does the dataset which corresponds to the administration of a significant burn injury to a rat animal model. This is due to the significant perturbation which has been administered to the system either in the form of a drug administration, or through a significant injury.

**Table 1:** The number of possible motifs vs. the number of motifs with one or more members for the five different datasets given the optimal parameter selections.

Dataset	Number of Possible Motifs	Number of Nonzero Motifs
Synthetic Dataset	19683	4918
Circadian Dataset	65536	3898
Acute Corticosteroid Dataset	19683	13320
Chronic Corticosteroid Dataset	177147	7180
Burn Dataset	1024	491
